# Validating the expression of miRNAs in healthy controls and SLE patients and to predict possible gene targets

**DOI:** 10.1186/1753-6561-9-S7-A4

**Published:** 2015-10-27

**Authors:** Amandeep Chugha, Siobhan Smith, Caroline Jefferies

**Affiliations:** 1 Department of Molecular and Cellular Therapeutics (MCT), Royal College of Surgeons in Ireland, Dublin, Ireland

## Background

Systemic Lupus Erythematosus (SLE) is a multisystem autoimmune connective tissue disease that has a strong female predominance (9:1) seen especially in women of child-bearing years [1]. Although the exact mechanisms of disease are not completely understood, the role of dysregulated microRNA (miRNA) has been implicated in the pathogenesis of SLE [2]. Clinically, the disease is unpredictable, with the current therapy for symptomatic patients associated with undesirable side effects and toxicity affecting multiple organs [3]. Therefore, in order to bridge the gap between disease and cure, an in depth understanding of disease pathogenesis is required to manufacture medications that tackle the underlying cause of the disease. In this study, we set out to validate differentially expressed miRNAs in SLE patient monocytes versus healthy controls, previously identified in a microRNA screen carried out in the group. Furthermore we aim to perform bioinformatics analysis to predict potential gene targets for these miRNAs that may play a role in the disease pathogenesis.

## Methods

RNA was extracted from monocytes from SLE patients and healthy controls previously collected in the laboratory. Bioinformatics analysis was undertaken to identify potential genes targeted by miRNAs of interest. The primers specific to the miRNAs were designed and optimised following which gene induction of miRNAs was determined by PCR. In order to investigate the expression of miRNAs, densitometric analysis of the gel electrophoresis was determined.

## Results

In the study, the expression of miRNA-107 and miRNA-132 were investigated. The expression of miR-107 was significantly greater in SLE patients than in healthy controls (p<0.00362), whilst there were no appreciable differences in miRNA-132 expression in SLE patients and healthy controls. SMURF1 and SOCS5 were identified as potential genes targeted by miRNA-107, however no significant change in expression in either genes were observed in SLE patients versus controls.

## Conclusions

There was a significant increase in the expression levels of miRNA-107 in SLE patients, while no significant changes in miRNA-132 was demonstrated. Further investigation into potential gene targets for miR-107 is required in order to understand the pathological importance of dysregulation of this miRNA in SLE and its therapeutic implications
